# Supplementary material for: Occurrence of Brucella ceti in striped dolphins from Italian Seas
Source: PLoS One. 2020 Oct 2;15(10):e0240178. doi: 10.1371/journal.pone.0240178 (PMC7531818; doi:10.1371/journal.pone.0240178)
Supplement: S1 Table — (DOCX) [file pone.0240178.s001.docx]

Supplementary Material

**Occurrence of *Brucella ceti* in striped dolphins from Italian Seas**

Giuliano Garofolo, Antonio Petrella, Giuseppe Lucifora, Gabriella Di Francesco, Giovanni Di Guardo, Alessandra Pautasso, Barbara Iulini, Katia Varello, Federica Giorda, Maria Goria, Alessandro Dondo, Simona Zoppi, Cristina Esmeralda Di Francesco, Stefania Giglio, Furio Ferringo, Luigina Serrecchia, Mattia Anna Rita Ferrantino, Katiuscia Zilli, Anna Janowicz, Manuela Tittarelli, Walter Mignone, Cristina Casalone, Carla Grattarola*

*** Correspondence:** carla.grattarola@izsto.it

**S1 Table. Full description of gross and microscopical findings, associated with complete diagnostic test results and the most likely cause of death for each case considered.**

| **Case #** | **Macroscopic findings** | **Histopathological findings** | ***Brucella* spp. investigation results** | **Ancillary diagnostic test results** | **Most likely cause of death** |
| --- | --- | --- | --- | --- | --- |
| **1.** | **External examination:** *Phyllobothrium delphini* encysted in subcutis of caudoventral abdomen (moderate parasitization) [45, 46]  **Lungs**: diffuse edema  **Spleen:** petechial haemorrhages  **Abdominal cavity:** *Monorygma grimaldii* encysted in ligaments of perianal region [45, 46]  **Stomach:** lack of ingesta  **CNS:** meningeal hyperaemia, cerebral edema | **Cerebrum:** non-suppurative leptomeningitis characterized by lympho-monocytic cells [47]. | A bacteriological examination allowed to isolate ***B. ceti* from CNS and spleen** [51, 52, 53]**.** | PCR for DMV antigen: negative (CNS)[48].  PCR for *T. gondii* antigen: negative (CNS)[49]. | Lesions at CNS level were consistent with *B. ceti* infection.  The stranding could have resulted from a severe cerebral impairment, associated to severe brain inflammation by *B. ceti*.  The isolation of the pathogen also at spleen level, associated to the moderate body condition of the striped dolphin (Table 1), the lack of ingesta and the moderate infection by larval cestode parasites, suggests a compromised host response. |
| **2.** | **External and internal examination:** no relevant gross pathological lesions  **Stomach**: lack of ingesta | **Cerebellum, cerebrum**: non-suppurative meningitis, characterized by lympho-monocytic cells [47]  **Spleen:** lymphoid depletion  **Lymph node:** lymphoid depletion **Liver:** hepatocyte vacuolar degeneration | Bacteriological examinations allowed to isolate ***B. ceti* from CNS** [51, 52, 53]. | PCR for DMV antigen: negative (CNS) [48].  PCR for *T. gondii* antigen: negative (CNS) [49]. | Lesions of the CNS were consistent with *B. ceti* infection.  The stranding could have resulted from a severe cerebral impairment, associated to severe brain inflammation by *B. ceti.* |
| **3.** | NE | NE | Bacteriological examinations allowed to isolate ***B. ceti* from CNS** [51, 52, 53]. | PCR for DMV antigen: negative (CNS) [48].  PCR for *T. gondii* antigen: negative (CNS) [49]. | Cause of death was not determined. |
| **4.** | **External exam:** NSLO  **CNS:** meningeal hyperaemia, cerebral edema | **Cerebellum, cerebrum:** severe, non-suppurative  meningo-encephalomyelitis, with a marked lympho-monocytic meningitis, more severe in medulla oblongata, characterized by lympho-monocytic plexocoroiditis and perivascular mononuclear cuffing | Bacteriological examinations allowed to isolate ***B. ceti* from CNS** [51, 52, 53]. | PCR for DMV antigen: negative (CNS) [48].  PCR for *T. gondii* antigen: negative (CNS) [49]. | Lesions of the CNS were consistent with *B. ceti* infection.  The stranding could have resulted from a severe cerebral impairment, associated to severe brain inflammation by a *B. ceti* infection. |
| **5.** | **External examination:** multifocal skin ulcers; *Phyllobothrium delphini* encysted in subcutis and *Monorygma grimaldii* encysted in muscle of caudoventral abdomen (moderate parasitization) [45, 46]  **Tongue**: focal ulcer  **Prescapular lymphnode**: lymphadenomegaly  **Lungs**: mild congestion, with rare subpleural calcified parasitic nodules  **Spleen**: splenomegaly  **Abdominal cavity**: *Monorygma grimaldii* encysted in peritoneal serosa [45, 46]  **Stomach:** scarce digested ingesta (squid beaks), correlated to a non-recent meal | **Cerebrum, cerebellum:** non-suppurative meningo-encephalitis**,** with mild and multifocal meningitis associate to mononuclear cells and plexochoroiditis; all brain section showed variable degrees of gliosis, perivascular edema, parasitic cysts, in presence of an inflammatory infiltrate composed of a large number of mononuclear cells.  At the level of the cerebellum a malacic area was evident, with gitter cells, protozoan cysts and perivascular cuffs [47]  **Lungs**: bronchointerstitial pneumonia  **Spleen:** lymphoid necrosis  **Prescapular lymph node:** lymphoid necrosis  **Pulmonary lymph nodes**: lymphoid necrosis  **Laryngeal tonsil:** lymphoid necrosis  **Liver:** multifocal necrotizing hepatitis  **Kidney:** interstitial nephritis | A bacteriological examination allowed to isolate ***B. ceti* from CNS, spleen and lung** (multiple-site infection), with negative results in prescapular lymph nodes and cerebrospinal fluid (CSF) [51, 52, 53].  The animal tested positive **by PCR for *Brucella* spp in CNS, lung, liver, spleen, prescapular, pulmonary and tracheobronchial lymph nodes**, with negative results in tongue ulcer, skin ulcer, laryngeal tonsil and CSF [54].  No evidence of anti-*Brucella* spp. antibodies was demonstrated in serum, cerebrospinal fluid and aqueous humor [24, 50]. | **PCR for DMV antigen: positive (CNS, bladder, pulmonary lymph node, skin ulcer)** [48].    **PCR for *T. gondii* antigen: positive (CNS, liver, skeletal muscle, pulmonary and**  **tracheobronchial lymph nodes, spleen)** [49].  **IHC for Morbillivirus antigen: positive (CNS, bladder)** [50].  **IHC for *T. gondii* antigen: positive (CNS)** [50].  **Anti-morbillivirus antibodies (1:8 - serum)** [50].  **Antì *T. gondii* antibodies (>1: 640 - serum), (1:160 - CSF), (1:80 - aqueous humor)** [50]. | Lesions of the CNS, characterized by multifocal meningoencephalitis with mononuclear cells, associated with multicentric lymphoid necrosis and multifocal necrotizing hepatitis, were consistent with a widespread *B. ceti* infection.  The stranding could have resulted from a severe cerebral impairment, associated to a coinfection involving DMV, *T. gondii*, and *B. ceti.* Noteworthy, in this case, a widespread *B. ceti* infection, detected by isolation and PCR in multiple tissues, associated to the absence of anti-*Brucella* spp antibodies and bacteriological and biomolecular negative results in CSF, suggest an acute fatal *Brucella* spp. infection**.** |
| **6.** | **External examination**: multifocal skin ulcers; *Phyllobothrium delphini* encysted in subcutis and *Monorygma grimaldii* encysted in muscle of caudoventral abdomen (moderate parasitization) [45, 46]  **Lungs**: severe parasitic bronchopneumonia by *Skrjabinalius guevarai* [45, 46]  **Abdominal cavity:** *Monorygma grimaldii* encysted in peritoneal serosa [45, 46]  **Stomach**: lack of ingesta  **Anterior stomach:** infestation by *Anisakis* spp. Nematodes [45, 46]  **Glandular stomach**: multifocal nodular granulomatous gastritis by *Pholeter gastrophilus* [45, 46] | **Cerebrum, cerebellum:** non-suppurative meningitis associated to a focal and mild encephalitis. A mild and multifocal meningitis with mononuclear cells was evident, more severe in in the occipital cortex. Rare perivascular cuffs of mononuclear cells were evident in the thalamus [47]. | A first bacteriological examination yielded negative results in spleen, lymph nodes, liver and lung.  A bacteriological examination performed at **CNS** level, after the detection of histopathological findings of neurobrucellosis, allowed to **isolate *B. ceti*** [51, 52, 53].  **The animal tested positive by** **PCR for *Brucella* spp in CNS.**  No evidence of anti-*Brucella* spp. antibodies was demonstrated in serum [24, 50]. | **PCR for DMV antigen: positive (CNS,** **bladder, lymph nodes, lung, liver, heart,** **spleen, kidney, muscle, skin)** [48].  PCR for *T. gondii* antigen: negative (CNS, lung, lymph nodes, liver, heart, spleen, muscle) [49].  Anti-morbillivirus antibodies: negative (serum) [50].  Antì *T. gondii* antibodies: negative (serum) [50]. | Lesions of the CNS were consistent with *B. ceti* infection.  The stranding could have resulted from a severe cerebral impairment, associated to a coinfection by DMV and *B. ceti*.  Based on PCR positive results for DMV, the multiorganic involvement observed suggests an immunocompromised host response. Considering the availability of histophatological data only at CNS level, further hypotheses on the pathogenic role played by DMV and *B. ceti* are not allowed, although a primary systemic DMV infection, followed by a subsequent acute fatal *B. ceti* infection, may represents a plausible option. |
| **7.** | **External examination:** multifocal skin ulcers; *Phyllobothrium delphini* encysted in subcutis and *Monorygma grimaldii* encysted in muscle of caudoventral abdomen (moderate parasitization) [45, 46]  **Lungs**: moderate parasitic bronchopneumonia by nematodes  **Lymph nodes**: lymphadenomegaly  **Abdominal cavity**: *Monorygma grimaldii* encysted in peritoneal serosa [45, 46]  **Stomach:** lack of ingesta  **CNS**: brain congestion with scattered hemorrhages | **Cerebrum**: severe non-suppurative  meningoencephalitis, associated to perivascular cuffs by mononuclear cells; severe mononuclear meningitis with rare polymorphonuclear cells; mononuclear inflammatory infiltrates were present in the choroid plexuses [47]  **Lungs**: moderate multifocal bronchointerstitial pneumonia  **Liver:** moderate hepatocyte vacuolar degeneration  **Heart:** mild multifocal myocarditis. | Bacteriological examinations allowed to isolate ***B. ceti* at CNS** level.  The animal tested negative for *Brucella* spp. in CNS, spleen, liver, lung, kidney, testes [51, 52, 53]. | **PCR for DMV antigen: positive (lung, liver, kidney);** negative (CNS, spleen, testes) [48].  PCR for *T. gondii* antigen: negative (CNS, liver) [49]. | Lesions of the CNS were consistent with *B. ceti* infection.  The stranding could have resulted from a severe cerebral impairment, associated to a coinfection by DMV and *B. ceti*.  Considering the presence of typical pathological changes, like multifocal bronchointerstitial pneumonia, suggestive of morbilliviral infection in an acute/subacute phase, associated to other findings that may reveal the effects of the viral infection, such as hepatocyte vacuolar degeneration and multifocal myocarditis, a primary systemic DMV infection, followed by a subsequent fatal *B. ceti* infection, could be plausibly hypothesized. |
| **8.** | **External examination:** *Phyllobothrium delphini* encysted in subcutis of caudoventral abdomen (moderate parasitization) [45, 46]  **Thoracic cavity:** hemothora**x**  **Lungs:** mild pulmonary edema associated to mild parasitic bronchopneumonia by nematodes  **Abdominal cavity**: hemoperitoneum  **Lymph nodes:** lymphadenomegaly  **Stomach:** lack of ingesta  **Anterior stomach:** moderate infestation by *Anisakis* spp. Nematodes [45, 46]  **Glandular stomach:** multifocal hemorrhagic gastritis  **Heart:** epicardial petechiae  **CNS:** meningeal hyperaemia, associated with brain congestion and cerebral edema | **Cerebrum**: non-suppurative meningitis,  associated to focal gliosis and perivascular cuffing [47]  **Prescapular lymph node**: lymphadenitis  **Lungs**: edema, congestion and monocytic broncho-alveolitis  **Liver:** hepatocyte degeneration and perivascular monocytic cuffing  **Intestine**: severe necrotizing monocytic enteritis | Bacteriological examinations allowed to isolate ***B. ceti* at CNS level**, with negative results in lung, liver, kidney, spleen and mesenteric lymph nodes [51, 52, 53].  Evidence of **anti-*Brucella* spp. antibodies** was demonstrated **in serum** [24, 50]. | **PCR for DMV antigen: positive (CNS)** [48].  PCR for *T. gondii* antigen: negative (CNS) [49]. | Lesions of the CNS were consistent with *B. ceti* infection.  The stranding could have resulted from a severe cerebral impairment, associated to a coinfection by DMV and *B. ceti*. |

CNS=Central Nervous System. NSLO=No significant lesions observed. NE=Not examined. CSF= cerebrospinal fluid
