# Supplementary material for: Occurrence of Brucella ceti in striped dolphins from Italian Seas
Source: PLoS One. 2020 Oct 2;15(10):e0240178. doi: 10.1371/journal.pone.0240178 (PMC7531818; doi:10.1371/journal.pone.0240178)
Supplement: Attachment — Submitted filename: Response to Reviewers.docx [file pone.0240178.s002.docx]

**Rebuttal letter**

We would like to thank you for giving us the opportunity to revise and resubmit this manuscript.

The manuscript has certainly benefited from the reviewers suggestions.

We have incorporated the suggested changes into the manuscript and we have extensively edited the manuscript in order to follow the suggestion of the reviewers in not making the descriptions of all the cases and giving more details on the ancillary tests.

To this end, we had a reorganization of the material methods sections and results creating also the supplementary table, in order to normalize the detail level of findings in the text.

To make changes easier to identify, we have included a version with all changes highlighted (“Revised Manuscript with Track Changes”), and the lines indicated in the point by point responses are referred to this document.

We have had one English speaker review the manuscript that has found this current version to be grammatically sound. I trust that the editing improved the manuscript.

**Editor requirements**

1. Please ensure that your manuscript meets PLOS ONE's style requirements, including those for file naming. The PLOS ONE style templates can be found at https://journals.plos.org/plosone/s/file? id=wjVg/PLOSOne_formatting_sample_main_body.pdf and <https://journals.plos.org/plosone/s/file?id=ba62/PLOSOne_formatting_sample_title_authors_affiliations.pdf>

*Done*

2. We note that you are reporting an analysis of a microarray, next-generation sequencing, or deep sequencing data set. PLOS requires that authors comply with field-specific standards for preparation, recording, and deposition of data in repositories appropriate to their field. Please upload these data to a stable, public repository (such as ArrayExpress, Gene Expression Omnibus (GEO), DNA Data Bank of Japan (DDBJ), NCBI GenBank, NCBI Sequence Read Archive, or EMBL Nucleotide Sequence Database (ENA)). In your revised cover letter, please provide the relevant accession numbers that may be used to access these data. For a full list of recommended repositories,see http://journals.plos.org/plosone/s/data-availability#locomics or <http://journals.plos.org/plosone/s/data-availability#loc-sequencing.>

*The WGS data is already submitted at NCBI GenBank and the data is visible to the editors and to the reviewers at the following link*  [*https://dataview.ncbi.nlm.nih.gov/object/PRJNA623338?reviewer=nccg8jpu5dp5m865md7u607b9i&archive=sra*](https://dataview.ncbi.nlm.nih.gov/object/PRJNA623338?reviewer=nccg8jpu5dp5m865md7u607b9i&archive=sra) *The data will be released and public upon the paper will be published*

3. In your Methods section, please provide additional location information of the study sites, including geographic coordinates for the data set if available.

*Done*

4. We note that you have stated that you will provide repository information for your data at acceptance. Should your manuscript be accepted for publication, we will hold it until you provide the relevant accession numbers or DOIs necessary to access your data. If you wish to make changes to your Data Availability statement, please describe these changes in your cover letter and we will update your Data Availability statement to reflect the information you provide.

*See the comment above*

5. We note that Figure 1 in your submission contain [map/satellite] images which may be copyrighted. All PLOS content is published under the Creative Commons Attribution License (CC BY 4.0), which means that the manuscript, images, and Supporting Information files will be freely available online, and any third party is permitted to access, download, copy, distribute, and use these materials in any way, even commercially, with proper attribution. For these reasons, we cannot publish previously copyrighted maps or satellite images created using proprietary data, such as Google software (Google Maps, Street View, and Earth). For more information, see our copyright guidelines: <http://journals.plos.org/plosone/s/licenses-and-copyright>.

*The figure 1 uses an i Italy map sourced OpenStreetMap contributors (https://www.openstreetmap.org/copyright/en) that can be used under a CC BY-SA as also reported in the figure1 legend*

**Reviewer #1**: **General comments**

**This is a very comprehensive study. However, some clarification is needed (see specific comments).**

**I would have like to see some comparison with MLVA results described in the manuscript. The PCR methods for identification could have been using another target (IS711) and/or be updated (qPCR).**

*In the present study we did not perform MLVA and we could not compare the whole SNP mapping with the MLVA. In the discussion section we commented that our findings agree with the findings of the B. ceti MLVA cluster from porpoise observed by Marquat. We wrote again the sentence to make the statement more clear. Now it reads:” Similar cluster partition was observed previously with MLVA data, which demonstrated the existence of the B. ceti group predominantly associated with porpoises “*

*We also agree with the reviewer about the use of more updated PCR protocols. Nonetheless, the study was conducted in several peripheral labs from the IZS network working mostly on cetacean stranding. The labs were not only organized for Brucella diagnosis. Consequently labs had different protocols and results on typing was confirmed by the Italian National and OIE reference laboratory for brucellosis. In the future we’ll harmonize the PCR tests using IS711 target and real-time tests that have more sensitive and specific diagnostic parameters.*

*Please see lines 873-875* *in Discussion section.*

**About pathology, the authors should discuss abortion (they seem not to have seen).**

*We thank the reviewer for the observation. To clarify this point, we add the sentence “No lesions were detected in the reproductive organs and no signs associated with a potential abortion were found in the only adult female sampled (case 6)” in the Discussion section.*

*Please see lines 758-759 in Discussion section.*

**The authors should mention that they did not sample in the Adriatic Sea. They should discuss that this may be one of the reasons why they did not report ST27**.

*Actually, as shown also in the figure 1 and table 1, we also had 1 case in the Adriatic Sea at the location of Otranto in 2013. It could be reasonable that, although this observation was only made based on 2 cases, the ST27 is restricted in the northern part of the Adriatic Sea (Croatia) while our strain belonged to the southern part of the Adriatic Sea. Nonetheless, the reason for presence of different lineages in the Adriatic Sea cannot be solved by our study because we probably need a more consistent sampling.*

**Specific comments**

**L61: lesions are described in cetaceans not in pinnipeds, so change “marine mammals” accordingly.**

*Done.* *Authors modified the text accordingly.*

*Please see line 70 in Introduction section.*

**L91: the first report on the isolation of B. ceti was in 1994. Do you mean first isolation in the Mediterranean sea?**

*Yes. Now it reads: “The isolation of B. ceti in the Mediterranean Sea was first achieved in 2009”*

*Please see line 116 in Introduction section.*

**L163: why were subcultures performed on Farrell’s media**

*We thank the reviewer for the observation. Our methodology foresee the subculture because if the Brucella broth is still heavily contaminated it is possible to see growth of colonies not related to Brucella spp. Thus, the SOP adopted reported to subculture the broths in selective media*.

*We revised the section Brucella isolation for clarity. Now it reads:*

*“For cases 1, 2, 3, 4, we used Farrell’s and Columbia blood Agar media, for casse 7 and 8 we added modified Thayer Martin and CITA media, while for cases 5 and 6 we used a combination of Farrell’s and CITA media. The solid media were incubated at 37°C, aerobically and in a microaerophilic atmosphere containing 5% CO2, for at least 10 days. Enrichment cultures were carried out in Brucella enrichment broth, supplemented with fetal horse serum and modified Brucella selective supplement, and incubated at 37°C in a microaerophilic atmosphere containing 5% CO2, while for cases 7 and 8 we added Thayer Martin broth. Weekly for six times or up until isolation, a loopful of the enrichment broth was streaked to Farrell’s Agar medium. Suspect colonies (circular, convex, shiny, 1-2 mm in diameter after 48-72 h) were seeded onto blood agar medium and incubated for a further 2 days before re-examination. When Brucella spp. was suspected based on the Gram’s staining [50], the colonies were tested for catalase, oxidase and urease activities [50]. Motility and slide agglutination tests with Brucella anti-A and anti-M antisera were also performed for cases 1, 2, 3, 4, 7, 8, together with nitrate reduction, H2S production and growth in the presence of CO_2_ for cases 5 and 6 [50].”*

*Please see lines 282-307 in Material and methods section.*

**L196: outer membrane of Brucella spp., not B. abortus. Why did you not use the IS711 based PCR or even better qPCR?**

*See the comment above.*

**L260: BAPS analysis (ref 57, 58) are not been used previously for Brucella. A short explanation is needed here.**

*BAPS analysis is based on Bayesian models used to infer the genetic structure within a population. The algorithm operates over allele frequency distributions (in our case SNPs) to predict whether there is an evidence of clustering within a dataset. HierBAPS expands the analysis further by iterating within specific clades in order to identify individual clusters.*

*To our knowledge, this method was published once previously for analysis of B. suis bv 2 population structure (Munoz et al., 2019, reference 60). For clarity, we modified the description of BAPS analysis in Material and Methods, which now reads: “**The population structure was assessed using a Bayesian approach implemented in BAPS 6.0 software with the module hierarchical BAPS (hierBAPS), which examines the existence of subgroups within a population and predicts the placement of individual sequences into specific clusters* [59, 60, 61]”.

*Please see lines 472-474 in Material and methods section.*

**L268-432: I am wondering if all the case descriptions are necessary. Perhaps Table 1, would be sufficient?**

*About this major point, we agree with the reviewers that providing a full description of all the cases could make the manuscript more difficult to follow. To satisfy the requests of both reviewers, we reorganized the “Results” and “material methods” sections in order to normalize the detail level of findings in the text. We removed the case descriptions and created a Supplementary Table 2 to include all the details about gross and microscopical findings, associated with complete diagnostic test results* *and the most likely cause of death for each case considered.*

*Please see:*

*lines 217-234; 282-307; 315-325; 411-414; 417-422 (Material and methods section);*

*lines 503-529; 552-564 (Results section);*

*Supplementary Table 2.*

**L463: which “other genomes?**

*We agree with the reviewer that the statement was too vague. We rephrased for clarity as follow:*

*“The publicly available B. ceti and B. pinnipedialis genomes were assigned to the STs 23, 24, 25, 26 and 27. “*

*Please see lines 566-567 in Results section.*

**L537-538: please explain how these results are in agreement with MLVA results.**

*In the present study we did not perform MLVA and we could not compare the whole SNP mapping with the MLVA. In the discussion section we commented that our findings agree with the findings of the B. ceti MLVA cluster from porpoise observed by Marquat. We wrote again the sentence to make the statement more clear. Now it reads:” Similar cluster partition was observed previously with MLVA data, which demonstrated the existence of the B. ceti group predominantly associated with porpoise”*

*Please see lines 873-875 in Discussion section.*

**L556-557: please be more specific. An association between exposure to pollutants and higher exposure/infection rates in marine mammals has been suggested (you touch upon these things later in the manuscript).**

*We agree with the reviewer that the statement “The role of environmental factors in this emerging disease remains elusive [36]”was not opportune, considering more recent published data about the association between exposure to pollutants and higher exposure/infection rates of infectious agents in marine mammals in general, also including specifically B. ceti (Davison et al 2011; Grattarola et al 2016).*

*For that, we delete the sentence opportunely, and we modified what expressed later in the manuscript adding these references.*

*Now it reads: “Moreover, further research is needed to understand the relationship(s) between the accumulation of persistent environmental pollutants in cetacean tissues and the effects of pollutant-related immunotoxicity on pathogenicity of B. ceti, as suggested elsewhere [16, 65].”*

*Please see lines 974-1038 in Discussion section.*

**L578: could you please explain to the prospective reader your multidisciplinary approach?**

*We thank the reviewer for the suggestion thus we tried to have a better explanation of the multidisciplinary approach. Now we stated as following:* *“Surveillance of strandings in Italy involves organisations from governmental and academic institutions with different areas of expertise such as public health, animal health and environment. Such network made our study possible, and our findings highlight the importance of the multidisciplinary approach in the monitoring of stranded cetaceans, with epidemiological data and laboratory information truly shared across sectors in the perspective of the one-health approach.”*

*Please see lines 1039-1043 in Discussion section.*

**Reviewer #2:**

The manuscript by Garofolo and coworkers entitled “Occurrence of Brucella ceti in striped dolphins from Italian Seas” describes the pathological analysis of 8 cases of stranding of striped dolphins in the coasts of Italy. Several diagnostic tools are used in order to investigate cause of stranding, including macro and microscopic observations, histology, serology, molecular detection, bacterial isolation, parasites identification in target tissues according to literature. In the presented cases, bacterial cultures were positive for Brucella and results confirmed by WGS.

The study is methodological sound and the analysis is thorough.

The way data is presented could be improved, particularly to improve focus as follows:

1. **Normalize the detail level of findings described. Although emphasis is given in Brucella ceti findings and characterization, there are other interesting findings related to the stranded dolphins that deserve attention. This should also be reflected in the materials and methods section, where description of protocols used to carry out the pathology study, parasites and worm identification as well as histological findings are need it.**

*We agree with the reviewer. We modified the “Materials and methods” and “Results” sections accordingly.*

*Specifically, authors modified the “Materials and methods” section, describing more in detail the protocols used, including the description of immunohistochemical investigations performed to explore the presence of DMV and T. gondii and the serological investigations preformed to assess the presence of specific antibodies for DMV and T. gondii.*

*Please see lines 217-234; 282-307; 315-325; 411-414; 417-422 (Material and methods section)*

*Moreover, authors reorganized the “Results” section in order to normalize the detail level of findings in the text, removing all the case descriptions now illustrated in the Supplementary Table 2 to include all the details about gross and microscopical findings,* *associated with complete diagnostic test results and the most likely cause of death for each case considered.*

*Please see lines 503-529; 552-564 (Results section) and Supplementary Table 2.*

**2. Remove description of cases and add it as a supplementary info. Description of each case can be improved connecting ideas instead of listing findings. Use table 1 to improve results section writing**

*See the comment above.*

**3. In the results section, indicate genome quality of the genomes analyzed, including depth coverage and percentage of mapping against reference genome. Were these criteria used in the selection of genomes to be included in the analysis.**

*We thank the reviewer for this question. In our study we tried to be as inclusive as possible without compromising on the quality of the analysed sequences. The lowest calculated depth in Italian dataset was 29X, while for majority of read sequences obtained from SRA the calculated depth exceeded 100X (with the minimum of 78 X). We did however limit our dataset to non-identical sequences and the sequences that mapped to the reference with less than 500 ambiguous matches.*

*Following the reviewer’s suggestion, we have now added a sentence in the Materials and Methods section: “Read coverage ranged from 29X to 113X, with an average of 63X.” And in the Results section: “The mapping of sequences to the reference ranged between 92 % and 99 %, with an average of 98 %.”*

*Please see lines 431-432 in Materials and Methods section and Lines 706-707 in Results section.*

**4. Several possible cause of deaths are suggested for the different cases. Indicate how the authors arrived to the different conclusions indicated for each case.**

*We thank the reviewer for this observation. Authors modified the “Discussion” section in order to clarify this point, introducing specific comments for cases investigated.*

*Now it reads:*

*“Considering the role of B. ceti infection in the striped dolphins under study, for cases 1, 2 and 4 the stranding could have resulted from a severe cerebral impairment, associated to severe brain inflammation by B. ceti.*

*Moreover, for case 2, the finding of a generalized lymphoid depletion, described before in dolphins with brucellosis [18, 62], suggests an immunocompromised host response, although the evidence of hepatocyte vacuolar degeneration could make additionally plausible the effects of toxic environmental pollutants and/or an undisclosed viral infection other than DMV.*

*The cause of death could not be hypothesized for case 3, considering the limited data available.*

*For cases 5, 6, 7 and 8 the stranding could have resulted from a severe cerebral impairment, associated to a coinfection by B. ceti and DMV and, for case 5, also T. gondii.*

*Noteworthy, for case 5, a striped dolphin in a poor body condition, the systemic spread of B. ceti infection, the evidence of additional Brucella-type lesions, including multicentric lymphoid necrosis and multifocal necrotizing hepatitis, associated to the absence of anti-Brucella spp antibodies and bacteriological and biomolecular negative results in CSF, suggests an acute fatal brucellosis infection that appears as the most likely contributing cause of death. DMV and T. gondii infections, associated to typical pathological findings, represented respectively by bronchointerstitial pneumonia and protozoan cysts at cerebral level, along with specific immunopositivity and presence of antibodies for both agents, may indeed support the potentially relevant role played by DMV and T. gondii in initiating the animal’s decline*

*Please see lines 769-845 in Discussion section.*

**5. Explain how the IonTorrent sequenced genomes were used in the presented work.**

*As described in Materials and Methods section, two strains of B. ceti isolated in Italy (10759 and 28753) were sequenced by our laboratory previously using Ion Torrent technology. These strains were used together with the other Italian strains and with the publicly available sequences to reconstruct B. ceti and B. pinnipedialis* *phylogeny based on the SNP analysis results. The assembled genomes were additionally used to determine MLST profiles in silico.*

**Minor points:**

**1. Introduction, page 10, lane 80: “The marine Brucella strains are classified into three major groups, with five clusters and 15 sequence 81 types (STs). Please explain what are the major groups and clusters and the context of this classification.**

*We modified the sentence for clarity now it reads: “According to Multi Locus VNTR (Variable copy Number of Tandem Repeats) Analysis (MLVA), the marine Brucella strains can be divided into three major groups containing eight clusters [28, 29], and the Multi Locus Sequence Typing (MLST) identified 15 sequence types (STs) so far as reported at the public PubMLST repository (https://pubmlst.org/brucella/).*

*Please see lines 103-106 in Introduction section.*

**2. Materials and methods, page 13, lane 152: “ The CNS of the case 6 was submitted to isolation retrospectively, since histopathology findings of neurobrucellosis had been observed”. Not sure what is the message from this sentence; retrospectively means that was carried out after a long time has passed since the histopathology analysis?**

*We thank the reviewer for the observation. We wrote again the sentence to make the statement more clear.*

*The isolation was not attempted after a long time has passed since the histopathology analysis. The CNS histopathological analysis was conducted late, compared to the post mortem examination.*

*Now it reads: “The CNS of case 6 was submitted for bacterial isolation subsequently to the observation of microscopic lesions suggestive of neurobrucellosis, thanks to the histopathological analysis performed retrospectively on CNS tissue of the animal under study.”*

*Please see lines 276-278 in Materials and Methods section*

**3. Materials and methods, page 13, lane 162: " The enrichment cultures in broth media were weekly subcultured (six subcultures) on Farrell’s Agar medium. Suspect colonies were tested for Gram stain reaction as well as for catalase, oxidase and urease activities, motility and slide agglutination tests with Brucella polyvalent antiserum.” Differences in smooth and rough phenotype have being described after several passages in B. ceti isolates. Please indicate on what subculture the agglutination test was performed and specifically what Brucella polyvalent antiserum was used.**

*We thank the reviewer for the observation because the subculture term was misleading. We revised the section Brucella isolation for clarity.* *Now it reads:*

*“For cases 1, 2, 3, 4, we used Farrell’s and Columbia blood Agar media, for cases 7 and 8 we added modified Thayer Martin and CITA media, while for cases 5 and 6 we used a combination of Farrell’s and CITA media. The solid media were incubated at 37°C, aerobically and in a microaerophilic atmosphere containing 5% CO2, for at least 10 days. Enrichment cultures were carried out in Brucella enrichment broth, supplemented with fetal horse serum and modified Brucella selective supplement, and incubated at 37°C in a microaerophilic atmosphere containing 5% CO2, while for cases 7 and 8 we added Thayer Martin broth. Weekly for six times or up until isolation, a loopful of the enrichment broth was streaked to Farrell’s Agar medium. Suspect colonies (circular, convex, shiny, 1-2 mm in diameter after 48-72 h) were seeded onto blood agar medium and incubated for a further 2 days before re-examination. When Brucella spp. was suspected based on the Gram’s staining [50], the colonies were tested for catalase, oxidase and urease activities [50]. Motility and slide agglutination tests with Brucella anti-A and anti-M antisera were also performed for cases 1, 2, 3, 4, 7, 8, together with nitrate reduction, H2S production and growth in the presence of CO2 for cases 5 and 6 [50].”*

*Please see lines 282-307* *in Material and methods section.*

**4. Materials and methods, page 15, lane 203: please specify the negative controls used. This is very important since the used PCR was not designed to detect Brucella specific amplicons in striped dolphins’ tissues.**

*We thank the reviewer for the observation. We specified the controls used in the text, accordingly.*

*Please see lines 317-318 in Material and methods section.*

**5. Discussion, page 27, first lane: “To our knowledge, this study represents the first survey of B. ceti infection in cetaceans of Italian waters and the first extensive characterization of B. ceti isolates reported to date”. Cetaceans is a general group of marine mammals. The study refers to a deep analysis of B. ceti isolates from striped dolphins.**

*We thank the reviewer for the observation. We modified the sentence accordingly.*

*Please see line 750 in Discussion section.*

**6. Discussion, page 27, lane 496 : “Considering the major gross and microscopical findings reported, many of the general pathological findings were not related to brucellosis.” This affirmation seems contradictory to the explanation that follows it. Please rephrase.**

*We agree with the reviewer and we thank him for catching the misleading sentence. We modified the sentence accordingly.* *Now it reads: “Although we observed several not pathognomonic signs, we detected specific gross pathological findings associated with Brucella infection, represented by hyperaemia of the meninges [9, 11] in three animals (cases 1, 4, 8).”*

*Please see lines 755-757 in Discussion section.*

**7. Discussion, page 28, lane 514: “Based on serological investigations, only one case (Case 8) of the three investigated (Cases 5, 6, 8) resulted positive for anti-Brucella spp. antibodies.” This observation deserves further discussion, since coinfections are relevant in the Mediterranean.**

**Why only one positive for RBT? Causes? Sample degradation? Bacteria with rough phenotype?**

*We thank the reviewer for the observation. Although in the authors’ opinion there is not enough sample size to draw conclusion, considering the two samples examined and resulted negative, the authors are confident that the quality of samples tested for case 5 did not alter the results: the striped dolphin examined was in a good conservation code, and serological test results for anti-morbillivirus and anti-T. gondii antibodies gave positive results.*

*For the other sample negative, belonging to case 6, also in a good conservation code, the negative results obtained also for anti-morbillivirus and anti-T. gondii antibodies suggests an immunocompromised host response, in an animal with a widespread DMV infection. We modified the sentence accordingly. Now it reads: “Interestingly, the anti-Brucella spp. antibodies observed in case 8 represent the only positive result among the three tested animals. The limited number of samples hampers any conclusion on the use of RBT for the detection of Brucella infection in dolphins. Nevertheless, the fact that the positive result was obtained by testing a fresh serum sample collected from an animal in a good conservation code, supports a true positive result [11]. Moreover, the negative serological findings suggest true negative results, likely supported by the simultaneous detection of other antibodies in case 5 and a supposed immunocompromised host response in case 6, in presence of a widespread DMV infection.”*

*Please see lines 846-853 in Discussion section.*

**How is this related with the fact that the authors state that Brucella appears to be acting as a secondary pathogen in lane 519? Brucella has been considered a primary pathogen in most host species. If the authors consider that this is not the case in striped dolphins, this should be properly discussed.**

*We thank the reviewer for the observation.*

*We delete the sentence, adding specific considerations about the pathogenic role of B. ceti.*

*We add these sentences:*

*“Considering the role of B. ceti infection in the striped dolphins under study, in cases 1, 2 and 4 the stranding could have resulted from a severe cerebral impairment, associated with severe brain inflammation caused by B. ceti infection. Moreover, in case 2, the finding of a generalized lymphoid depletion, described before in dolphins with brucellosis [18, 62], suggests an immunocompromised host response, though the evidence of hepatocyte vacuolar degeneration could additionally make the effects of toxic environmental pollutants and/or an undisclosed viral infection other than DMV plausible.*

*The cause of death could not be hypothesized in case 3, considering the limited data available.*

*In cases 5, 6, 7 and 8 the stranding could have resulted from a severe cerebral impairment, associated with a coinfection by B. ceti and DMV and, for case 5, also T. gondii. Noteworthy, in case 5, a striped dolphin in a poor body condition, the systemic spread of B. ceti infection, the evidence of additional Brucella-type lesions (multicentric lymphoid necrosis and multifocal necrotizing hepatitis), with the absence of anti-Brucella spp antibodies and negative bacteriological and biomolecular results in CSF, suggests an acute fatal brucellosis infection that appears to be the most likely contributing cause of death. DMV and T. gondii infections, associated with typical pathological findings, represented respectively by bronchointerstitial pneumonia and protozoan cysts at cerebral level, along with specific immunopositivity and the presence of antibodies for both agents, may indeed support the potentially relevant role played by DMV and T. gondii in initiating the animal’s decline.”*

*Please see lines 769-845 in Discussion section.*

**8. Discussion, page 28, lane 521 and following: “The highest frequency of B. ceti infection was confirmed in juveniles (6 out of 7 cases with age determined), followed by adults (1 out of 7).” Comparison with the data from Brazil are not accurate since sample population is different, taking into account several cetacean species. A more accurate comparison can be done by comparing only striped dolphin’s data in the Brazil study, and recent Costa Rica and US studies.**

*We thank the reviewer for the observation, and we reorganized the sentences according to the observations.*

Now it reads:” *The highest frequency of B. ceti infection was confirmed in juveniles. This observation seems to be in discordance with a study performed in striped dolphins stranded in Costa Rica [62], in which meningoencephalomyelitis was revealed in the same number of juveniles and adults that displayed neurological syndromes before death, as well as with a previous study in marine mammals stranded in Brazil [18]. Specifically, in a population made up of several cetacean species, the highest frequency of Brucella spp. infection was confirmed in the newborn calves, whereas, within the genus Stenella, the most commonly infected age-group were the adults.”*

*Please see lines 853-863 in Discussion section.*

Additional revisions apported:

- *Authors modified the affiliations*

*see lines 7-8 and 20-22*

- *Authors modified the sentence “Since 2007, isolates of Brucella spp. from marine mammals have been further classified into two species, B. ceti and B. pinnipedialis that infect cetaceans and pinnipeds, respectively, on the basis of molecular methods, differences in metabolism and host-bacteria interaction [5]” in “Since 2007, isolates of Brucella spp. from marine mammals have been classified further based on molecular investigations, differences in metabolism and host-bacteria interactions, into two species, B. ceti and B. pinnipedialis that infect cetaceans and pinnipeds, respectively [5].”*

*see lines 67-69*

- *Authors modified “Animals and samples included in the study” in “Dolphins”*

*see line 163*

- *Authors modified the legend of Fig. 1. “Stranding sites of striped dolphins infected by B. ceti under study, Italy, 2012-2018. Geographical mapping was obtained by ArcGIS® software by World Topographic Map by Esri using the geographical coordinates found from the single strandings. (For interpretation of the references to colour in this figure legend, the reader is referred to the web version of this article).” in*

*“Fig. 1. Stranding sites of striped dolphins infected by B. ceti under study, Italy, 2012-2018. Geographical mapping was obtained by ArcGIS® software using the geographical coordinates found from the strandings.*

*Location data (dots):*

*Case 1 dark green: Gallipoli Lido Pizzo, Apulian coastline, Ionian Sea (39.58 N, 18.00 E) March 11th 2012*

*Case 2 purple: Porto Cesareo Bacino Grande, Apulian coastline, Ionian Sea (coordinates not available) October 2nd 2012*

*Case 3 yellow: Alimini, Otranto, Apulian coastline, Adriatic Sea (coordinates not available) November 13th 2013*

*Case 4 light blue: Porto Cesareo Bacino Grande, Apulian coastline, Ionian Sea (coordinates not available) December 9th 2014*

*Case 5 green: Savona, on the Ligurian coastline, Ligurian sea (44.94 N, 8.18 E) February 15th 2017*

*Case 6 red: Ardore, on the Calabrian coastline, Ionian Sea (38.11 N, 16.12 E) May 12th 2017*

*Case 7 pink: Maruggio, on the Apulian coastline, Ionian Sea (40.17 N, 17.35 E) December 6th 2017*

*Case 8 blu: Manduria, on the Apulian coastline, Ionian Sea (40.18 N, 17.40 E) November 5th 2018*

*The Italy map was used under a CC BY-SA copyright from OpenStreetMap contributors (https://www.openstreetmap.org/copyright/en) (For interpretation of the references to colour in this figure legend, the reader is referred to the web version of this article).*

*see lines 179-200*

- *Authors added a sub-section* “*Necropsy and diagnostic investigations”*

*see line 210*

- *Authors modified the list of cases investigated by molecular detection of Brucella spp from tissues, and changed the sentence” Specifically, molecular detection was attempted on CNS, spleen, lung, liver, pre-scapular, tracheobronchial, pulmonary lymph nodes, tongue and skin ulcers, laryngeal tonsil and CSF of Case 5 and on CNS of case 6.” in “ Specifically, molecular detection was attempted on CNS, spleen, lung, liver, pre-scapular, tracheobronchial, pulmonary lymph nodes, tongue and skin ulcers, laryngeal tonsil and CSF of case 5; on CNS of case 6; on CNS, spleen, liver, lung and testes of case 7.”*

*see lines 315 and 319-321*

- *Authors removed the sub-section “Histopathological analysis”, putting the content in the sub-section “Necropsy and diagnostic investigations”*

*lines 218-221*

- *Authors added data about immunohistochemical and serological investigations performed about DMV and T. gondii infection diagnosis*

*see lines 226-231*

- *Authors modified the sub-section “Serological tests” in “Serological tests for Brucellosis”, changing the content from “ Dolphin sera, cerebrospinal fluids or aqueous humor were collected when available (Cases 5, 6 and 8), kept frozen at -20°C and tested by rapid serum agglutination (RBT) using RBT antigen produced from B. abortus strain S99 [24, 51] to detect anti-smooth Brucella spp. antibodies. For serum of case 8 the test was performed on fresh sample “ in “ Serum, cerebrospinal fluid and aqueous humor from case 5, and serum from cases 6 and 8, were tested by rapid serum agglutination (Rose Bengal plate test, RBT) using RBT antigen produced from B. abortus strain S99 [24, 48] to detect anti-smooth Brucella spp. antibodies. For case 8, the test was performed on a fresh sample, while for cases 6 and 8 was carried on thawed sera. “*

*see line 410 and 411-414*

- *Authors removed the sub-section “DNA extraction”, putting the content, integrated, at the beginning of the sub-section “Whole genome sequencing and bioinformatics”*

*lines 417-422*

- *Authors apported minor revisions to Table 1, mainly about formatting style*

*lines 517-530*

- *Authors modified the sentence “The data obtained by whole genome SNP analysis suggest an interesting relationship between phylogeny and geographical distribution of B. ceti strains in Italy, worthy of further investigations, including strains not yet available and strains of future isolation.” in The data obtained by whole genome SNP analysis suggest an interesting relationship between phylogeny and geographical distribution of B. ceti strains in Italy, therefore providing new insights into the phylogenetic structure of B. ceti in the Mediterranean. Nevertheless, further studies from the Mediterranean Sea are required to elucidate the molecular evolution of B. ceti and its actual distribution.”*

*lines 949-953*

- *Authors modified the sentence “Given the demonstrated zoonotic potential of this bacterium [31, 32, 33], proper handling of stranded animals and safety management of necroscopic and diagnostic investigations are strongly recommended to avoid the risk of transmission to humans.” in “Finally, based on the demonstrated zoonotic capability of B. ceti [31, 32, 33], proper handling of stranded animals, together with an ad hoc adoption of all necessary biosafety and biosecurity measures and protocols during post mortem and diagnostic investigations on stranded cetaceans are strongly recommended to avoid the risk of transmission to humans of this and other zoonotic pathogens.”*

*lines 1046-1050*

- *Authors modified the section Author Contributions, including Giovanni di Guardo in the sub-section Investigations*

*line 1059*

- *Authors added the references:*

*Pintore MD, Mignone W, Di Guardo G, Mazzariol S, Ballardini M, Florio CL et al. Neuropathologic findings in cetaceans stranded in Italy (2002-14). J Wildl Dis. 2018 Apr;54(2):295-303. doi: 10.7589/2017-02-035*

*Muñoz PM, Mick V, Sacchini L, Janowicz A, De Miguel M J, Cherfa MA et al. Phylogeography and epidemiology of Brucella suis biovar 2 in wildlife and domestic swine. Vet Microbiol. 2019; 233: 68-77.*

*Davison NJ, Perrett LL, Law RJ, Dawson CE, Stubberfield EJ, Monies RJ et al. Infection with Brucella Ceti and High Levels of Polychlorinated Biphenyls in Bottlenose Dolphins (Tursiops Truncatus) Stranded in South-West England. Vet Rec 2011 Jul; 2;169(1):14. doi: 10.1136/vr.d2714.*

*lines 1258-1260; 1308-1310; 1325-1328*

Minor revisions were also performed at lines 31, 43, 47, 51, 62, 66, 73-74, 77, 80-81, 85, 101, 107-108, 111-112, 122-124, 144, 148-149, 159, 322, 327, 309-310, 719-723, 727, 729, 752-754, 760, 763-764, 766-768, 864, 866-869, 871, 875-876, 877-881, 884-885, 955-957, 959, 962, 963-965, 966-968, 969-971.
