# Supplementary material for: Occurrence of Brucella ceti in striped dolphins from Italian Seas
Source: PLoS One. 2020 Oct 2;15(10):e0240178. doi: 10.1371/journal.pone.0240178 (PMC7531818; doi:10.1371/journal.pone.0240178)
Supplement: Attachment — Submitted filename: Response to Reviewers.docx [file pone.0240178.s003.docx]

**Rebuttal letter**

We would like to thank you for giving us the opportunity to revise and resubmit this manuscript.

The manuscript has certainly benefited from the reviewers suggestions.

We have incorporated the suggested changes into the manuscript and the Supplementary Table 2 in order to follow the suggestions of the reviewers.

The lines cited are referred to the “Manuscript” file.

**Reviewer #1**:

**Reviewer #1: I have 1 comment that perhaps need an explanatory sentence and reference in the final manuscript.**

**Serology:**

**The RBT has limitations when used in wildlife species, particularly marine mammals. therefore ELISAs are excellent alternatives. More, a "universal" indirect ELISA for detecting antibodies in mammals, including marine mammals has been described and documented: Nymo I.H., Godfroid J., Asbakk K., Larsen A.K., das Neves C.G., Rødven R., Tryland M. 2013. A protein A/G indirect enzyme-linked immunosorbent assay for the detection of anti-Brucella antibodies in Arctic wildlife. J. Vet. Diagn. Invest., 25: 369-375. doi: 10.1177/1040638713485073.**

**I feel that reference to ELISA is needed. I trust the RBT results reported in this study. However, "true" result may be misleading. Indeed, infected animals may be classified negaltive by RBT but positive by iELISA. So, a word of explanation is needed here.**

*We thank the reviewer for the suggestion, we modified the text accordingly. Now it reads:*

*”The limited number of samples hampers any conclusion on the use of RBT for the detection of Brucella infection in dolphins. Nevertheless, the fact that the positive result was obtained by testing a fresh serum sample collected from an animal in a good conservation code, supports a true positive result [11]. Moreover, the negative serological findings are supported by the simultaneous detection of other antibodies in case 5 and a supposed immunocompromised host response in case 6, in presence of a widespread DMV infection. RBT test, while generally considered consistent, may produce false results due to variety of factors [65]. Therefore, in order to screen the immunological status of the examined animals, other serological tests such as ELISA could be used, as previously shown [66]. Although some discrepancies between results of RBT and ELISA tests have been reported, ELISA tests, such as iELISA have been successfully used to detect anti-Brucella antibodies in odontocetes and arctic wildlife [65, 66] and could therefore serve as a complementary method serological response to B. ceti in dolphins.”*

**Additional comment about pollution and immunocompromition:**

**Actually, the link is difficult to demonstrate beyond a general statement.**

**Nymo I.H., das Neves C.G., Tryland M., Bårdsen B.J., Santos R.L., Turchetti A.P., Janczak A.M., Djønne B., Lie E., Berg V., Godfroid J. 2014. Brucella pinnipedialis hooded seal (Cystophora cristata) strain in the mouse model with concurrent exposure to PCB 153. Comp. Immunol. Microbiol. Infect. Dis., 37: 195-204. doi: 10.1016/j.cimid.2014.01.005.**

**So, a word of caution could be needed.**

*We thank the reviewer for the observation. We wrote again the sentence to make the statement more appropriate.* *Now it reads:*

*Moreover, a detailed understanding of the effects of pollutant-related immunotoxicity on pathogenicity of B. ceti, as suggested by some case reports (16, 69), is required, particularly in light of conflicting result obtained using ex vivo model (70).*

Line 470.

**Reviewer #2**:

**Reviewer #2: This version of the manuscript is a good improvement from the previous version. There are some issues that I think are important to address, related mainly with the description of methods used. This can help to reproduce data by independent researchers.**

**Materials and Methods section**

**Move:**

**252All strains isolated from the striped dolphins under study were identified asassigned to B. ceti using**

**253 the PCR-RFLP method [51149] and then subjected to genomic analysis at the OIE and National and**

**254 OIE Reference Laboratory for Brucellosis, Istituto Zooprofilattico Sperimentale dell’of Abruzzo e**

**255 del and Molise, Teramo, Italy.**

**To the WGS section and rephrase accordingly, assuming that the same DNA extraction method was used.**

*Revised accordingly. The DNA extraction was moved at the line 204.*

**261 The reactions were loaded as previously reported using B. suis bv 2 strain Thomsen and no template**

**262 control as positive and negative controls, respectively**

**No clear to this reviewer what this means. Please specify what was added in a no template control as positive and what is a negative control under this context. A proper negative control should include a sample from a similar animal or tissue from the same animal where no isolation of Brucella was obtained. No evidence of standardization of this PCR for cetacean tissues is given, therefore, this control is essential.**

*We regret that the sentence was unclear but in the statement we reported the use of B. suis bv 2 strain Thomsen as positive control and no template control for the negative control. Typically, a negative control for PCR is one sample that should not give amplicons, therefore, any visible bands might be a result of contamination or non-specific amplification. The lab works under ISO/IEC 17025 regulation and has validation procedure for the diagnostic tests therefore the PCR was analytically validated for detecting Brucella DNA.*

*Moreover using tissue samples from bacteriological negative animals could be misleading because there is the possibility of finding dead cells or free DNA from Brucella that gives you positive PCR reactions and negative bacteriological isolation. This situation could be even more evident using tissue from the same animal where you may find positive bacteriological results from one tissue and negative bacteriological from another one.*

*We revised for clarity the line 219. Now it reads: “The reactions were loaded as previously reported using B. suis bv 2 strain Thomsen as positive control and no template control as negative control.”*

**As stated by the authors in their response, please include the following info the related to selection criteria of the public genomes used in the study:**

**“We did however limit our dataset to non-identical sequences and the sequences that mapped to the reference with less than 500 ambiguous matches.”**

*As suggested, we inserted the following sentence in Materials and methods section:*

*“The dataset was limited to non-identical sequences that mapped to the B. ceti reference genome with less than 500 ambiguous matches (GenBank Accession Numbers NC_022905.1; NC_022906.1)”*

*Line 259.*

**According to the answer given by the authors:**

**"As described in Materials and Methods section, two strains of B. ceti isolated in Italy (10759 and 28753) were sequenced by our laboratory previously using Ion Torrent technology."**

**And a quick database search, it seems that these sequences were already published. If that is the case, please refer properly.**

*Revised accordingly, citing the reference 42:*

*Ancora M, Marcacci M, Orsini M, Zilli K, Di Giannatale E, Garofolo G, et al. Complete Genome Sequence of a Brucella ceti ST26 Strain Isolated from a Striped Dolphin (Stenella coeruleoalba) on the Coast of Italy. Genome Announc. 2014 Mar 6;2(2). pii: e00068-14. doi: 10.1128/genomeA.00068-14.*

*Line 257.*

**Results section**

**First lane: “Post-mortem and histopathological investigations were performed on seven positive animals”**

**I assume it means** **seven of the eight animals with positive culture for Brucella?**

*Yes. We revised for clarity the line, specifying appropriately.*

*Line 278.*

**Supplementary Table 2**

**Please add references for all methods used, particularly those used for parasites**

*We thank the reviewer for the suggestion. We modified the Supplementary Table 2 accordingly, and we added the references for methods used for parasites in the Section “Necropsy and diagnostic investigations”:*

*Line 161.*
